# Differing impacts of the COVID-19 pandemic on farmers and intermediaries: insights into the Ecuadorian cocoa value chain

**DOI:** 10.1186/s40100-024-00302-0

**Published:** 2024-02-15

**Authors:** Guillermo Zambrano, Lina M. Tennhardt, Moritz Egger, Karen Ramírez, Adriana Santos, Byron Moyano, Michael Curran

**Affiliations:** 1 Escuela Superior Politécnica del Litoral, ESPOL, Facultad de Ciencias de la Vida, Campus Gustavo Galindo Km 30.5 Vía Perimetral, P.O. Box 09-01-5863, Guayaquil, Ecuador; 2 Research Institute of Organic Agriculture (FiBL), Frick, Switzerland; 3 Georges Lemaître Earth and Climate Research Centre, Earth, and Life Institute, University of Louvain, Ottignies-Louvain-la-Neuve, Belgium

**Keywords:** Cocoa, Ecuador, COVID-19, Smallholder-farmers, Intermediaries, Supply chain resilience

## Abstract

The COVID-19 pandemic generated diverse impacts and responses in agricultural value chains worldwide. Cocoa is a key crop for Ecuadorian exports, and the analysis of effects the pandemic had on value chain actors contributes to the understanding of their individual capacities to coping with a major shock. The purpose of this study was to assess the number and severity of impacts and responses implemented by two links in the cocoa value chain to the pandemic, based on a survey of 158 cocoa farmers and 52 cocoa intermediaries from the main cocoa-producing provinces of the northern coast of Ecuador in 2021. Surveyed farmers and part of the intermediaries form part of the sustainability program of a large Swiss chocolate manufacturer. The impacts and responses reported were grouped into seven resources according to the Activity System Approach. Then, a comparison between groups was applied using the Wilcoxon rank sum test for nonparametric data, determining the most severe impacts and effective resilience responses among the actors. The results reveal that farmers and intermediaries were similarly affected by the pandemic, reporting 21 and 16 negative impacts, respectively. Farmers experienced a higher number and severity of impacts on financial and social resources, while intermediaries on human and material resources. The strongest impact was the loss of sales, reported by 65% of farmers and 58% of intermediaries. Farmers implemented more social responses that they judged highly effective, while intermediaries implemented more human responses that they judged highly effective. Public policy should enhance the social resources of farmers by strengthening their associativity and the capacities of their members, as mechanisms to mitigate their vulnerability to future health and climate crises. The financial resources of both actors should be protected through public credit and agricultural insurance.

## Introduction

The COVID-19 pandemic severely impacted various sectors of the global economy. Globalized food systems and value chains have shown to be susceptible to disruption in trade routes and limited labor availability (Béné 2020). The COVID-19 pandemic has disrupted agricultural value chains in their dynamics of production, transportation, logistics, and dietary choices of consumers, among others (Roubík et al. 2022; Sridhar et al. 2022; Stephens et al. 2022; Štreimikienė et al. 2022). However, at the international level, it became evident that agricultural systems did not collapse completely in the face of the pandemic due to the declarations of governments that the food sector is essential and needs to continue its operations despite the mobility restrictions imposed for other sectors (Béné et al. 2021). Arita et al. (2022) argue that this lower impact was also due to the low-income elasticity of food demand.

Export-oriented farmers located at the upstream end of those value chains are often considered especially vulnerable to socioeconomic shocks as they depend on other value chain actors (Ansah et al. 2019). This vulnerability has been demonstrated by the impacts cocoa farmers suffered as a consequence of the COVID-19 pandemic on a global scale, like loss of sales (Fountain and Hütz-Adams 2020), international shipping delays (Kumar and Jolly 2021), and subsequent loss of cocoa quality (Cadby 2021). In Ecuador, the situation for cocoa farming communities was further exacerbated by reduced access to inputs (Mena and Gutiérrez 2021) and rising inequalities among community members (Córdoba et al. 2021). Due to the vulnerability to unforeseen shocks of upstream actors like cocoa farmers faced with the impacts of the COVID-19 pandemic, strengthening the resilience of agricultural systems has become a cornerstone of international organizations like the FAO (2023) or OECD (2020). Small-scale or subsistence farming systems showed resilience to the effects of changes in prices and market conditions due to the cultivation of various foods for self-consumption and the preponderance of family labor in agricultural work. However, the economic income of households showed a decrease mainly in those with off-farm income affected by mobility restrictions (Workie et al. 2020; Lopez et al. 2021; Roubík et al. 2022). Strategies applied by small-scale farmers in Central America and Mexico to cope with the loss of income include an increase in direct sales to consumers, incorporation of family labor returning from the cities into agricultural work, and collective and solidarity agricultural and marketing activities (Lopez et al. 2021).

The COVID-19 pandemic provided numerous reasons to reflect on the vulnerabilities of modern agricultural systems (Darnhofer 2020). Dixon et al. (2021) identified that productive diversification, shorter and more flexible value chains, and greater integration with information and communication technologies were relevant factors in promoting the resilience and sustainability of farming systems. Rasul (2021) indicated that creating more interactive environments between various actors in the value chains and promoting research and innovation to generate pandemic-and-climate-smart food systems is necessary to face these shocks with high resilience.

The pandemic also generated lessons learned about technological and social innovations in value chains, which can be replicated in future crises that affect the sustainability of farming systems. Reported innovations that countered the impacts of the COVID-19 pandemic range from industry 4.0 technologies, big data and blockchain to remote work, digital commerce, as well as circular and collaborative economy (Sarkis 2021). The countries of the Andean Community and Central America (e.g., Ecuador, Peru, and Dominican Republic) have historically stood out for their production of specialty cocoa, but at the same time they have been strongly affected by epidemics, pests, diseases, and natural phenomena that have influenced cocoa producers, affecting their livelihoods. Consequently, the generation of information on the mechanisms used by farmers to cope with the pandemic is a valuable input to mitigate the impact of future global crises (Cadby 2021). In this context, the study of the resilience of the actors in Ecuador's cocoa value chain to the pandemic represents a valuable source of information to improve the dissemination of social innovations that promote sustainable development.

The concept of resilience that emerged in the 1970s out of studies on system ecology (Holling 1973) is becoming increasingly popular in studies on the susceptibility of food systems against disturbances (Folke et al. 2010), like the COVID-19 pandemic. Although substantial challenges exist to transferring ideas of ecological systems to agency-driven socioeconomic systems (Darnhofer 2014), a multitude of operational approaches has been developed in recent years (Tukamuhabwa et al. 2015; Quinlan et al. 2016; Serfilippi and Ramnath 2018a; Meuwissen et al. 2019). Those approaches generally aim to promote the resources (e.g., social, economic, natural, human, material, etc.) necessary to keep the systems functioning while maintaining the individual needs of actors affected by a disturbance (Cabell and Oelofse 2012).

Nevertheless, there is still no consensus about the definition and measurement criteria required to analyze the resilience of food systems (Folke et al. 2010; Béné et al. 2014; Darnhofer 2021a; Kaldjob 2021). Approaches for resilience assessment at the farm level often refer to concepts of adaptive cycles derived from systems ecology (Meuwissen et al. 2019) or define resilience by its relation to the concepts of sustainability and vulnerability (Serfilippi and Ramnath 2018b). Furthermore, it is widely agreed upon that farm-level resilience can be understood as the combination of three response capacities: absorption, adaption, and transformation (Folke et al. 2010; Cabell and Oelofse 2012; Béné et al. 2014; Darnhofer 2014, 2021b; Béné and Doyen 2018; Meuwissen et al. 2019). Therefore, we understand the resilience of farming systems as a dynamic process that goes beyond the passive attributes of a system to cope with shocks by engaging in active responses (Béné et al. 2014) through adaptation and transformation, potentially leading to entirely new system configurations (Darnhofer 2021a).

In the context of entire value chains, the concept of resilience is orientated to provide a framework for enhanced risk management (Pettit et al. 2019). Building resilience for value chains is about enabling the value chain actors to respond as quickly and efficiently as possible to changes in the marketplace and ensure the continued delivery of goods and services along the value chain (Jain et al. 2017). The focus of resilience in value chains lies in maintaining a primary system state and imposes higher relevance on the importance of cost-effectiveness than in the scientific debate about the resilience of farming systems (Tukamuhabwa et al. 2015). In the value chain context, resilience enhancements are assumed to require substantial investments into multiple value chain actors that may never pay back or produce additional costs if the corresponding disruption never occurs, limiting willingness to engage in resilience action (Pettit et al. 2019). However, there also exist efforts to reframe agricultural supply chain resilience concepts by emphasizing the socioeconomic importance of upstream actors (Aboah et al. 2019).

Previous studies have predominantly concentrated either on evaluations of farm-level resilience (Jacobi et al. 2015; Wongnaa and Babu 2020) or entire supply chain evaluations (Adobor 2020; Hamidu et al. 2022), but studies that give insights into and compare the implemented resilience strategies of different supply chain actors are rare. Given the distinct conceptualizations and definitions of resilience, depending on the focus on the farm level or supply chain, conflicts and trade-offs seem imminent when trying to align efforts to counter a disturbance. Nevertheless, it may yield interesting results to compare farm-level resilience with downstream supply chain resilience, as individual supply chain actors' efforts to enhance their resilience may negatively or positively affect the goals of other members in the supply chain (Aboah et al. 2021). In an effort to bridge farm level and supply chain resilience concepts, we aim to compare how upstream actors are affected and respond to the impacts of the COVID-19 pandemic in relation to downstream actors by establishing evidence on engaged resilience strategies and resource mobilization. Furthermore, this process should yield valuable information on the compatibility of farm-level and supply-chain resilience conceptualizations.

By using the Ecuadorian cocoa sector as a case study, the present study aims to answer the following research questions: (1) How did the impacts of the COVID-19 pandemic differ between cocoa producers and intermediaries, and (2) What kind of responses were mobilized by cocoa producers and intermediaries to counteract the effects of the COVID-19 pandemic? We followed the hypotheses that farmers experienced more severe impacts and were able to mobilize less-effective responses to counter the effects of the COVID-19 pandemic than intermediaries due to their lower economic opportunities and lower position in the value chain. The second hypothesis implies that the pandemic generated more significant impacts and responses in the financial resources of both actors due to the alteration in commodity prices in the early stages of the pandemic, as well as in human resources, considering the restriction and biosecurity measures imposed by governments.

The cocoa value chain was selected due to its importance in the economy in Ecuador and its social impact. Since 2014, cocoa production in the country has been growing at an average annual rate between 12 and 15% (Avadí et al. 2021; Avadí 2023; MAG 2023). Currently, Ecuador is the third largest exporter and producer of dry cocoa beans worldwide and one of the first suppliers of fine-flavor cocoa in the international market along with the Dominican Republic and Peru (ICCO 2023). Cocoa exports (dry beans and derivatives) represent 4.8% of non-oil exports, and contribute 6.9% to agricultural gross value added (MAG 2023). The cocoa production process, which covers work in the field until export, generates 397,502 sources of direct and indirect work, where 76% is occupied by farmers and their families (MAG 2023).

To answer the research questions, a survey was conducted in 2021, two years after the initial outbreak of the COVID-19 pandemic. The main cocoa-producing provinces of Ecuador were selected to obtain data from both farmers and intermediaries about the perceived impacts and responses implemented in the face of the effects of the pandemic in the production units, allowing for a comparison between both groups of actors under study. This research represents an input for informed decision-making by local and international actors that promote the development of Ecuador's cocoa value chain, generating information in relation to the COVID-19 pandemic about: (a) identification of the most vulnerable actors, (b) capacities and strategies implemented by the actors, and (c) impacts on the resources available to the actors to face the shocks.

This manuscript first provides a detailed overview of the COVID-19 impacts on the case study, followed by a description of the methodological approach. Then, the results are presented and discussed in light of our research questions and existing literature before providing some concluding remarks.

## Materials and methods

### Case study description

Ecuador has had a strong orientation toward cocoa production since colonial times. In fact, cocoa bonanzas have positioned it as one of the main exporters worldwide, driving the development of its economy (Acosta 2012). These economic dynamics have had historical and cultural implications in the producing areas, influencing the structuring of social classes in the ancient Ecuadorian coast, some of whose characteristics are still evident today. Between the late nineteenth and twentieth centuries, cocoa production in the country was mainly concentrated in large farms owned by economically powerful families, who also invested cocoa profits in the commercial and banking sector in the city of Guayaquil, the main port connecting Ecuadorian cocoa with the world. Economic crises, investment in new crops, and political decisions have contributed to the reconfiguration of cocoa production. Currently, more than 80% of cocoa production is in the hands of smallholder farmers (Abad et al. 2020).

Cocoa plays an important role in the country's agricultural production, given that in 2021 it was the crop with the largest planted area (626,962 ha), producing 302,904 mt. Ecuador has experienced notable increases in yields in the last decade (Hütz-Adams et al. 2022), from 0.37 in 2010 to 0.56 mt/ha in 2021 (MAG 2022); according to Kozicka et al. (2018) and Fountain and Hütz-Adams (2018), state support programs and crop renewal contribute to this growing trend. The highest productive concentration of cocoa has been in the coastal provinces, considering this crop's high agroecological and economic aptitude (Sánchez et al. 2019). By 2021, the main cocoa-producing provinces were Los Ríos (28% of dry beans production), Guayas (24%), Manabí (15%), and Esmeraldas (12%) (MAG 2022).

Cocoa production in Manabí is characterized by a predominance of fine-flavor cocoa (i.e., *Cacao Nacional*), which in some studies is estimated to reach between 65.50% (Barrera et al. 2018) and 85% (MAG 2018) of the planted area. The main marketing channel used by smallholder farmers is retail intermediaries (72.28%) and agricultural associations (17.09%) (Barrera et al. 2018). The 95% of the cocoa produced in Manabí is exported. In the province of Los Ríos, *Cacao Nacional* is also highlighted for its high quality. The main marketing channel is intermediaries, who, together with exporters, obtain relatively the highest economic benefits for the quality of the product (Morales et al. 2018; Ibarra 2019). However, unlike Manabí and Esmeraldas, the cocoa variety CCN-51 (the Spanish acronym for *Colección Castro Naranjal No 51*) predominates in Los Ríos with 58% of the planted area compared to *Cacao Nacional* (Guilcapi 2018). At the national level, the trend is to switch to the CCN-51 variety, considering its higher profitability and tolerance to diseases, even though it does not have the floral aroma and flavor characteristics of the *Cacao Nacional* (Ramírez et al. 2022).

In Ecuador, 98% of cocoa production is generated on plots of less than 20 ha., grouping 185,000 farmers. On the other hand, intermediation is carried out by more than 4,600 actors, most of which collect the cocoa and carry out the fermentation and drying process, obtaining price and product quality advantages over farmers (Avadí et al. 2021). The location and distance of the farm from the collection centers also influence the price paid to the producer (Sánchez et al. 2019). Once harvested, most of the cocoa coming from different provinces is transported to the port of Guayaquil for export. The cities of Guayaquil and Durán have become increasingly attractive for national and international export companies, as the production and quality of Ecuadorian cocoa generate good conditions for the formation of clusters dealing with cocoa and its derivatives (Ramírez et al. 2022).

Ecuador consumes about 2% of its total cocoa production (Arvelo et al. 2016), demonstrating an important orientation toward world markets. In 2021, the main form of export was cocoa beans (US$819 million), and its main destination market was the USA (US$216 million); the EU bloc received 28.29% (US$ 265 million) of Ecuador's cocoa and cocoa derivatives exports (ITC 2022a, b, c). The 2010–2021 time series reflects an increasing trend of Ecuadorian exports despite the pandemic, registering an increase of 20.4% between the years 2019 and 2020. Prices paid to the producer per quintal (= 45.36 kg) of cocoa in the country experienced their largest drop in the month of March 2020 (arrival of COVID-19 to the country), standing at US$53.55/quintal (US$1.18/kg), presenting a decrease of 51% compared to the previous month (MAG 2022). Prices recovered and averaged US$91.54/quintal (US$2.02/kg) from May to December 2021 (MAG 2022).

At the beginning of the pandemic, the measures imposed by the Ecuadorian government were based on mobility restrictions, mandatory use of biosecurity equipment (e.g., masks and alcohol), and prohibition of social gatherings. The measures were imposed at the national level and controlled through epidemiological traffic lights (Ministerio de Salud Pública de Ecuador 2022). The medical, food, and export sectors were exempt from mobility restrictions to carry out their work (Servicio Nacional de Gestión de Riesgo y Emergencias 2020), which contributed to the mitigation of the impacts of the pandemic. In the period 2021–2022, the measures implemented were based on strengthening the vaccination process. In order to increase the coverage of the vaccination plan, the Ecuadorian army in coordination with the Ministry of Public Health promoted the Fénix plan. For this purpose, the army planned the mobilization of 3000 soldiers and logistical resources to provide support to more than 800,000 Ecuadorians living in rural areas, peripheral zones and isolated communities, thus attempting to apply a comprehensive response to the pandemic, creating community epidemiological monitoring centers, installing vaccination centers, and promoting biosecurity measures (Parlamento Andino 2021).

### Sampling and data collection

**Fig. 1:**
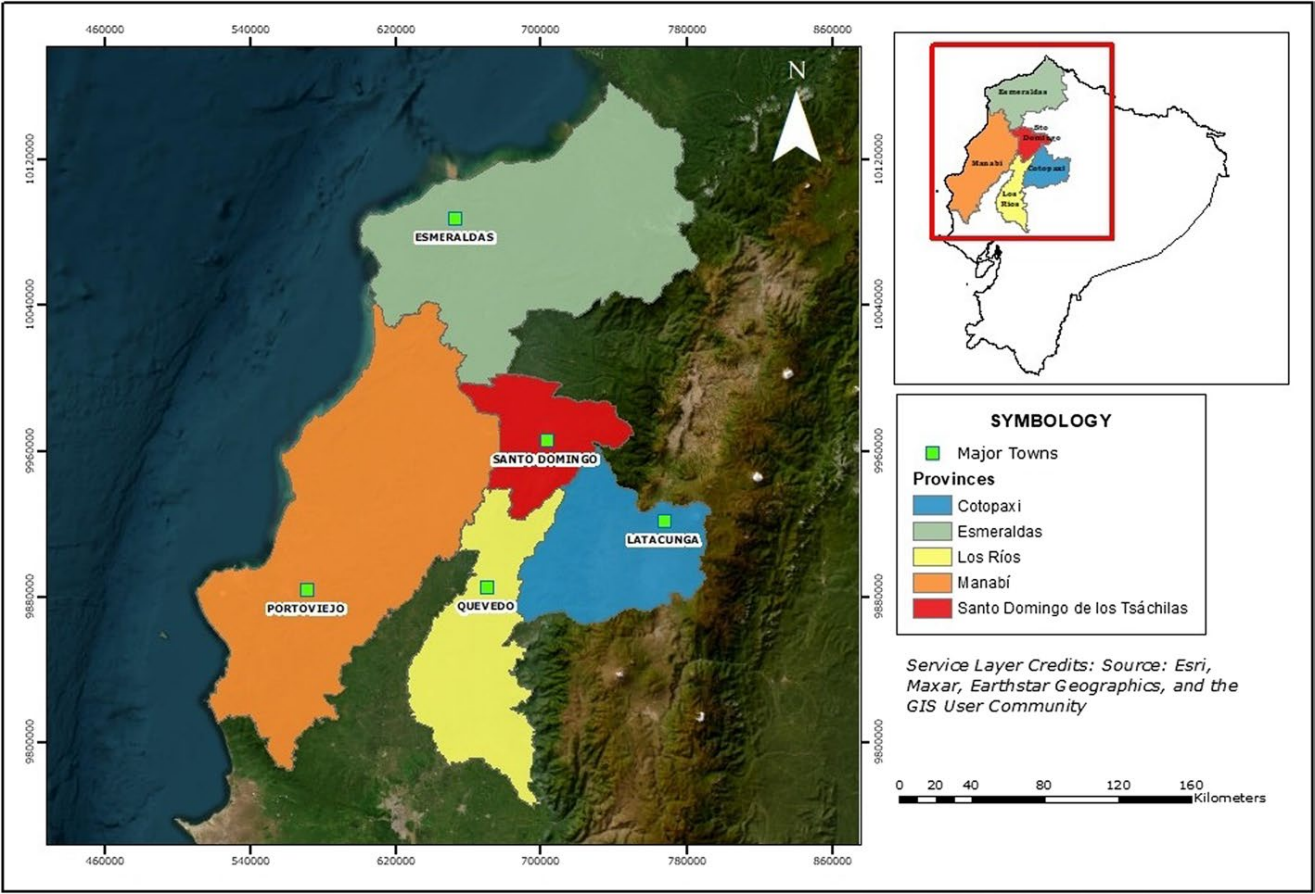
Study area

Farmers and part of the intermediaries in our sample form part of the in-house sustainability program of a large Swiss chocolate manufacturer. Program farmers typically sell their cocoa to program intermediaries, who then again sell to a multinational trading company that delivers the cocoa beans to the Swiss chocolate manufacturer. The trader is also in charge of implementing the sustainability program, which includes traceability ambitions, farmer training, distribution of in-kind premiums, and community development activities (for more information, see Tennhardt et al. 2022). Figure 1 shows the Ecuadorian provinces in which the primary data were collected.

From > 6000 program farmers, six intermediary groups were selected in three provinces in north-western Ecuador and then randomly selected subsamples of 17–28 farmers, in total 158 (Table 1). The sampling approach for intermediaries differed. On the one side, it consisted of the six program intermediaries belonging to the sampled farmer groups. On the other side, convenience sampling was implemented for additional interviews with random cocoa intermediaries based on accessibility to the data collection team. The sample of interviewees was a result of feasibility within the project framework.

**Table 1:** Sample sizes for farmers and intermediaries by province

Province	Farmers	Intermediaries
Manabí	86	16
Cotopaxi	22	4
Los Ríos	0	10
Esmeraldas	50	17
Santo Domingo	0	5
Total	158	52

Trained enumerators and part of the authors interviewed farmers and intermediaries using a structured questionnaire between August and September 2021. Participation was voluntary, and informed consent was obtained orally from respondents at the beginning of each interview for the collection and processing of personal data in an anonymized way. Particular care was paid to the mode of data collection during the organization of interviews due to existing fears of a COVID-19 infection. Therefore, all participants were offered an interview by phone, instead of a face-to-face meeting, which 22 farmers preferred. Data collection was performed in accordance with all relevant institutional and national ethical guidelines and received ethical approval by the FiBL Department of Food System Sciences under reference number FSS-2022-001.

### Indicator selection

The questionnaire was divided into two parts. The first part contained open-ended questions about the impacts of the COVID-19 pandemic and responses the interviewees engaged in to counter the consequences of the pandemic. The aim of this approach was to get unbiased impressions from the interviewees without suggesting potential responses beforehand. Severity scores ranging from 1 to 5 were collected for each impact mentioned, and effectiveness scores in the same range were recorded for the responses reported by the interviewees. This procedure allowed us to not only analyze the quantity of reported impacts and responses but also to account for the quality of those observations.

The second part of the questionnaire contained closed-ended statements with 5-point Likert scales for approval, including 21 statements for farmers and seven for intermediaries. The extent and time requirement for intermediary questionnaires was reduced as much as possible to increase the response rate and thus included fewer statements. At the time of survey creation in early 2021, only limited information about the impacts of the COVID-19 pandemic on the Ecuadorian cocoa sector was published in peer-reviewed journals. We oriented the selection of indicators around theoretical frameworks of farm-level resilience (Cabell and Oelofse 2012; Serfilippi and Ramnath 2018b; Meuwissen et al. 2019) while ensuring enough flexibility to account for potential indicators that may be unique to the conditions resulting from the COVID-19 pandemic. Integrated impacts that were reported in the literature at the time of survey creation ranged from the loss of sales (Fountain and Hütz-Adams 2020; Teye and Nikoi 2021) to limited access to supplies and inputs (Mena and Gutiérrez 2021), lack of labor (Teye and Nikoi 2021), transportation delays (Kumar and Jolly 2021), loss of cocoa quality (Cadby 2021), and increasing community inequality (Córdoba et al. 2021), among others. Additional statements were adapted from a flash poll on COVID-19 and its impact on small chocolate businesses by the FCCI (2021).

Finally, basic information on farms and farming households was collected. However, no background information was collected for intermediaries to reduce their time investment and increase the response rate, considering the intermediaries' anonymity regarding the operation of their business models.

### Data analysis

Data analysis for this study followed four steps. First, each respondent group’s share of impacts and responses was calculated with descriptive results for severity and effectiveness to provide a general overview of impacts, their severity, responses, and their effectiveness mentioned by farmers and intermediaries. Second, the impacts reported by farmers and intermediaries were grouped into seven resources: financial, human, informational, material, natural, identity and social, using the Activity System approach (Gasselin et al. 2020). This approach allows the analysis of the interacting activities of a social entity and the available resources that are mobilized in a social and in agroecological context. In this case, this approach was useful for understanding the decision-making process and the interactions of different resources faced during the COVID-19 pandemic.

The third step consisted of weighing each impact and response with the corresponding severity and effectiveness score. The perceived severity and effectiveness of impacts and responses include high value for estimating respondents' real experiences. It is argued that experiencing several impacts with low severity might affect a farm or intermediary equally than one highly severe impact. Finally, to compare weighted impacts and responses between farmers and intermediaries in the seven resource categories and Likert-scale responses, a Wilcoxon rank sum test was used for nonparametric data.

All statistical analyses were performed in R (vers. 4.1.0, R Project for Statistical Computing, RRID:SCR_001905), via RStudio (vers. 2022.02.01 + 461, RStudio, Q19 RRID:SCR_000432). All graphs were created using the ggplot2 package in RStudio; the map was created using ArcGIS (www.esri.com, vers. 10.8). Data and code are available here: https://figshare.com/s/04c583d9b718af9ca0b0.

## Results

### Descriptive results

Interviewed farmers were mostly male (76%), with an average age of 53 years (Table 2). They managed on average 13.7 ha of land, of which 36% were devoted to cocoa production. Almost 41% of farm managers generated off-farm income, mainly working as a laborer on other farms.

**Table 2:** Mean (sd) and share of farmer characteristics

	Farmers (*n* = 158)
Farm manager age (years)	53.25 (13.95)
Male farm manager (%)	75.9%
Farm manager married/civil union (%)	82.7%
Formal education of farm manager (years)	8.14 (4.20)
Household size (# members)	3.69 (1.53)
Farm size (ha)^a^	13.70 (17.12)
Cocoa plot size (ha)^a^	5.04 (3.76)
Farm managers generating off-farm income (%)	40.7%

^a^Does not include total sample

The group of intermediaries interviewed had cocoa buying and selling points located near the villages and on the main roads connecting the cantonal capitals. Two types of intermediaries were interviewed: (1) stockpilers with their own or rented shops, who had a larger infrastructure to store or dry cocoa, and (2) stockpilers without the infrastructure to store cocoa, who only weigh and buy cocoa to resell it to other larger intermediaries. Depending on the location of collection points, some intermediaries additionally collected other products such as corn, soybeans, rice, and coffee.

### Impacts on farmers and intermediaries

**Fig. 2:**
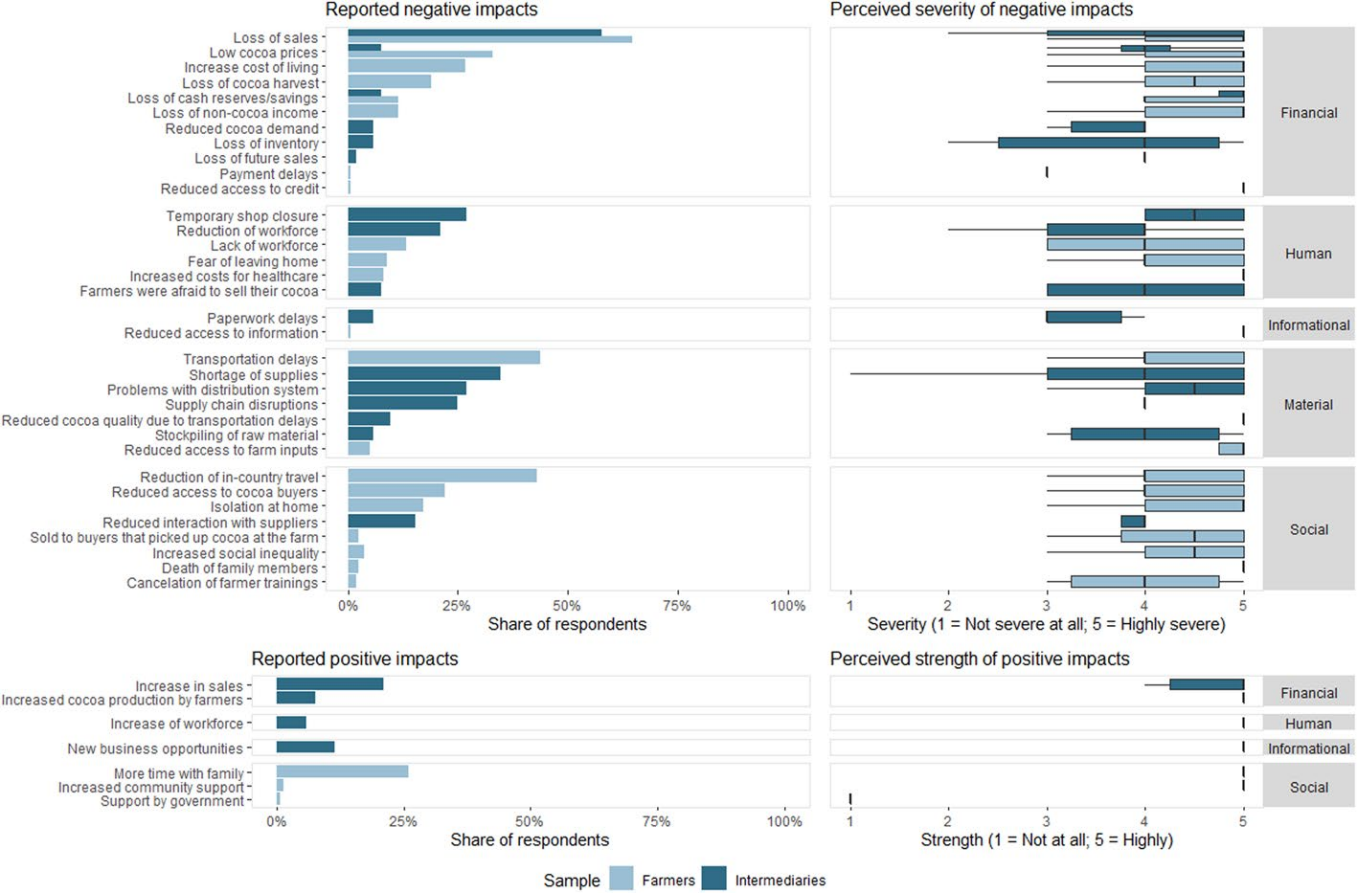
Reported positive and negative impacts and their severity and strength by farmers and intermediaries

The open-ended question on the experienced impacts due to the COVID-19 pandemic resulted in 24 impacts mentioned by farmers and 20 by intermediaries (Fig. 2). Out of these, 21 and 16 impacts, respectively, were negative. Both farmers and intermediaries experienced most negative impacts on their financial resources. By far the strongest impact for both groups was “loss of sales,” reported by 65% of farmers and 58% of intermediaries. 33% of farmers additionally mentioned “low cocoa prices” as a substantial economic impact. Both farmers and intermediaries rated the severity of experienced negative impacts as medium to high, with median values between 3.0 and 5.0 on a 5-point Likert scale.

Both farmers and intermediaries also reported positive impacts. Farmers only mentioned positive impacts on their social resources, mainly spending “more time with family” (26% of respondents). The strongest positive impact for intermediaries was “increase in sales” mentioned by 21% of the sample. All positive impacts were rated as strong, except for the experienced “support by the government” (median of 1.0). In the rural areas of some of the cantons under study, interviewees identified that the return of migrants from the cities and the constant search for commercial opportunities generated the opening of new businesses to serve the communities, such as grocery stores, pharmacies, and community banking correspondents, among others. These perceptions were captured in the field notes of the enumerators.

### Responses by farmers and intermediaries

**Fig. 3:**
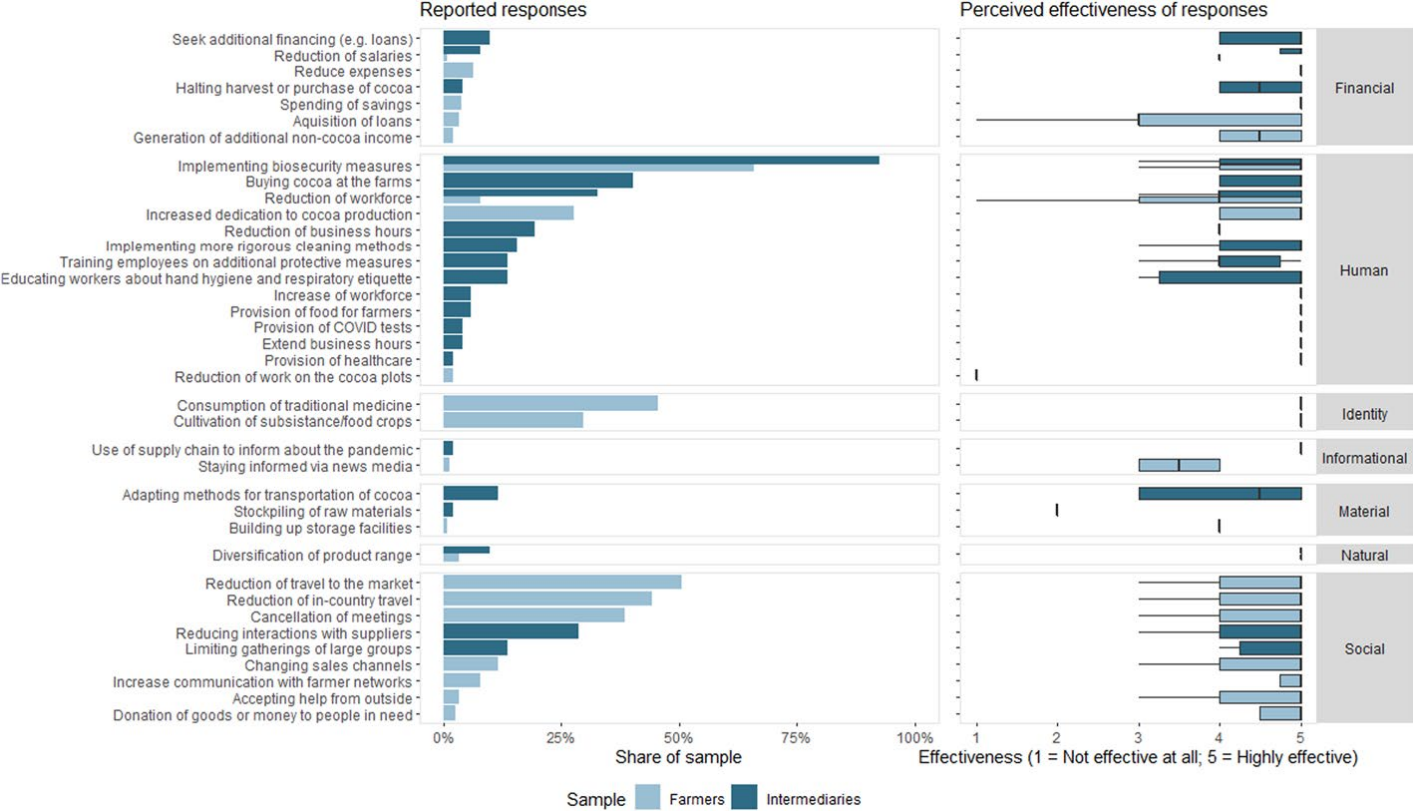
Reported responses and their effectiveness by farmers and intermediaries

The open-ended question on implemented responses to the COVID-19 pandemic resulted in 21 responses mentioned by both farmers and intermediaries (Fig. 3). Intermediaries' responses to counter the impacts of the pandemic mainly activated human resources, with 12 responses mentioned. They mainly reported to “implement biosecurity measures” (92% of intermediaries) and “bought cocoa at the farm” (40% of intermediaries), representing a change in their purchasing channels. The challenges posed by the pandemic also created the need to incorporate other commercial strategies into the business models of intermediaries, such as stocking new agricultural products and providing inputs and food as an incentive to attract farmers' produce and take advantage of the high international demand for cocoa and other food products.

Farmers rather responded to the pandemic within their social resources, with seven responses mentioned. These mainly included social distancing and isolation, including a “reduction of travel to the market” (51% of farmers) and a “reduction of in-country travel” (44% of farmers). Responses among identity, informational, material, and natural resources were mentioned less often, the “consumption of traditional medicine” (46% of farmers) and “cultivation of subsistence and food crops” (30% of farmers) being the most frequent responses within their identity resources. The effectiveness of responses was rated mostly medium to high with median values of 3.0–5.0 on a 5-point Likert scale. Only “stockpiling of raw materials” (intermediaries) and “reducing work on cocoa plots” (farmers) were perceived less effective with median values of 2.0 and 1.0, respectively.

### Comparison of impacts and responses

**Fig. 4:**
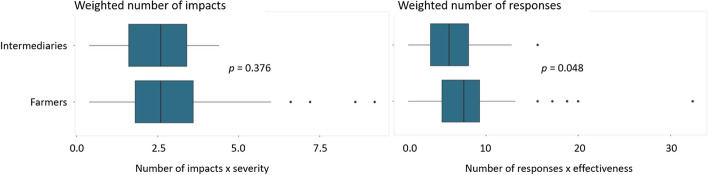
Statistical comparison of means: weighted number of impacts and responses by sample group

Comparing weighted impacts between farmers and intermediaries shows that both groups were similarly affected by the pandemic with respective mean values of 2.8 and 2.4 (Wilcoxon rank sum test, *p* = 0.376; Fig. 4). Farmers, however, reported more and more effective responses than intermediaries, with respective mean values of 7.6 and 6.5 (Wilcoxon rank sum test, *p* = 0.048).

**Fig. 5:**
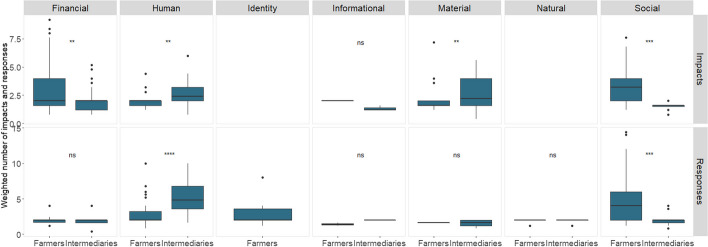
Weighted number of impacts and responses by sample group in the seven resource categories

Dividing the impacts by resource category shows similar results for informational resources (Wilcoxon rank sum test, *p* > 0.05; Fig. 5), and the responses show similar results for financial, information, material, and natural resources (Wilcoxon rank sum test, *p* > 0.05). However, farmers experienced significantly more and stronger financial and social impacts (Wilcoxon rank sum test, *p* < 0.01) and implemented more and more effective social responses than intermediaries (Wilcoxon rank sum test, *p* < 0.001). On the contrary, intermediaries experienced significantly more and stronger human and material impacts (Wilcoxon rank sum test, *p* < 0.01), yet also applied significantly more and more effective human responses than farmers (Wilcoxon rank sum test, *p* < 0.001).

### Impact statements by farmers and intermediaries

**Fig. 6:**
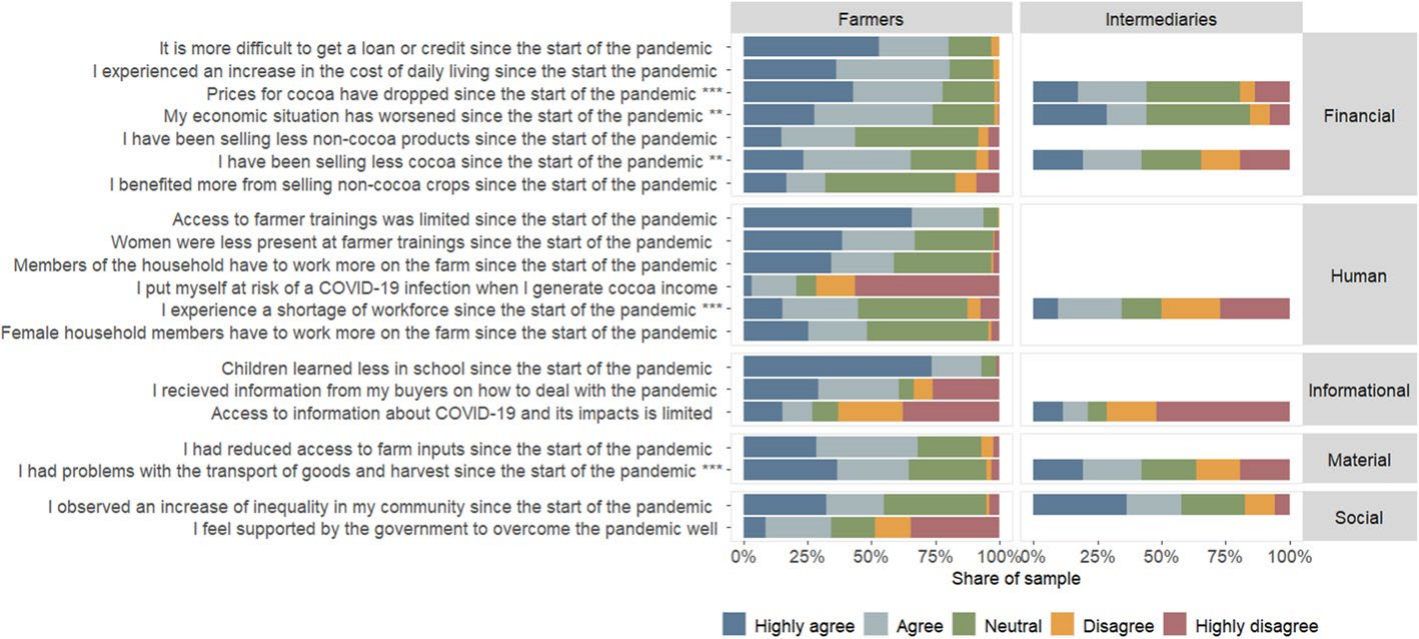
Overview of respondents' agreement with statements regarding the impact of the COVID-19 pandemic Statistical comparison of responses between group using Mann–Whitney U test (** = *p *< 0.01; *** = *p *< 0.000)

The closed-ended questions on respondents' level of agreement with different statements showed a diverse picture (Fig. 6). At least 50% of farmers highly agreed or agreed to five out of seven financial impacts, with highest agreement to the statements on “difficulties to get a loan or credit” and “increased cost of daily living” since the start of the pandemic, to which 80% agreed/highly agreed. Responses varied significantly by respondent group: “Prices for cocoa have dropped since the start of the pandemic” and “My economic situation has worsened since the start of the pandemic,” for example, received > 75% of agreement by farmers, yet < 50% of agreement by intermediaries (Wilcoxon rank sum test, *p* < 0.001 and = 0.007, respectively). Furthermore, > 80% of farmers agreed/highly agreed to the statement that they “benefited more from non-cocoa crops for household consumption.”

Agreement to statements by farmers regarding human resources was more varied. Some statements received a high degree of agreement, for example, 93% that “access to farmer training was limited” since the start of the pandemic, yet contrarily, 71% of farmers disagreed/highly disagreed with the statements that they “put themselves at risk of a COVID-19 infection when generating cocoa income.” Both farmers and intermediaries showed a high level of disagreement/high disagreement with “access to information about COVID-19 and its impact is limited” (63% and 71%, respectively; Mann–Whitney U test, *p* = 0.110). While 64% of farmers and 42% of intermediaries agreed/highly agreed that they had “problems with the transportation of goods and harvest” among the material resources, responses varied significantly between groups (Wilcoxon rank sum test, *p* < 0.000).

## Discussion

This study set out to assess the differences in impacts experienced and responses implemented by cocoa farmers and intermediaries in Ecuador in light of the current COVID-19 pandemic and national responses.

### Cocoa producers and intermediaries were differently impacted by the COVID-19 pandemic

The results show that both farmers and intermediaries experienced mostly negative impacts due to the pandemic, which was to be expected (Fig. 2). Globally, the crisis caused by the pandemic disrupted value chains and agri-food systems, resulting in negative economic effects such as the loss of jobs and farmers' livelihoods (Clapp and Moseley 2020; Kumareswaran and Yugantha 2022). The results also validate one of the hypotheses since both actors had greater impacts on their financial resources (Fig. 3), as they were negatively affected by the fall in cocoa prices and sales, mainly during the first months of the pandemic. Social and human resources were also affected, considering mobility restrictions and biosecurity measures. Statistical tests of means showed that both actors had a similar number of impacts and intensity (Fig. 4). However, a significant difference was found between the resources that were impacted, as farmers experienced a higher number and severity of impacts on financial and social resources, while intermediaries on human and material resources (Fig. 5).

Regarding financial resources, most farmers felt highly impacted economically by the COVID-19 pandemic, with lower cocoa prices and fewer marketing opportunities (Fig. 3). In comparison, most intermediaries felt fewer economic shocks and partly even reported higher cocoa prices. In several developing countries, there was also evidence of strong impacts on agricultural and non-agricultural income and access to food for rural communities due to the fall in prices paid to producers and difficulties in accessing markets, particularly those with more restrictive sanitary measures (Ogisi and Begho 2021; Hammond et al. 2022). The effectiveness and inclusiveness of public social assistance policies during the pandemic were also lower in developing countries, considering that there was a positive correlation between GDP per capita and these criteria (Saha et al. 2022).

At the macro-level, in Ecuador, cocoa export data reflected a growing trend compared to the pre-COVID-19 period. Cocoa exports as raw material in US$ had year-on-year percentage increases of 22.40% (2019–2020) and 0.54% (2020–2021). The net weight of exports, in mt, had a year-on-year percentage change of 19.13% (2019–2020) and 1.58% (2020–2021) (MAG 2022). Despite these higher economic revenues for the country's trade balance, the study results reveal that farmers had a higher perception of deterioration of cocoa prices and their economy compared to the intermediaries due to the pandemic. The distribution of economic benefits and the impacts in the financial resources of farmers should be analyzed in light of the pre-existing structural conditions in the cocoa value chain, given that intermediaries have greater negotiation power to obtain substantial economic advantages (Avadí et al. 2021). This disadvantage is amplified as intermediaries are one of the main marketing channels (MAG 2019) and, in some coastal cantons, are sources of financial capital for farmers (Ramírez et al. 2022).

Social resources mostly concern social distancing and isolation (Fig. 3), which farmers often reported. Farmers had high fear of contracting the disease and then being unable to receive medical treatment. Experiences of losing family members and friends to COVID-19 increased these fears. This behavior was observed in several rural areas of Ecuador reflecting the limited state investment in public health and the difficulty for rural families to access medical care (FIAN ECUADOR et al. 2020). Although the public mitigation policies of several governments of developed and developing countries focused on expanding social assistance programs during the pandemic, it has become evident that several vulnerable populations, mainly in rural areas, were not effectively included in the programs, so the design of these policies to face future crises should focus on criteria of equity and inclusion (Saha et al. 2022; Tan et al. 2023).

Globally, governments, as part of their pandemic containment policies, declared the agricultural sector as essential and granted less mobility restrictions to allow value chains to function (OCDE 2021). In the case of intermediaries in Ecuador, although there was free mobility as part of a strategic export sector (Servicio Nacional de Gestión de Riesgo y Emergencias 2020), they had to adhere to biosecurity measures dictated by local authorities and were impacted due to disruptions in the provision of supplies in the value chain. Intermediaries were also affected in their commercial operations because COVID-19 infected workers were unable to work, forcing some collection points to reduce public attention time and temporarily close, which strongly impacted business cash flows. Considering that 79.9% of the economically active population in rural areas of the country works in the informal sector and that 43.4% of the country's workers live in households without access to social security, the pandemic deepened the vulnerability of farmers and employees of intermediaries' businesses, where high informality is evident (OCDE 2020; INEC 2023). In the case of African countries, the pandemic had a strong effect on the informal economy, where several workers in this sector experienced a drastic decrease in their income (Rukasha et al. 2021).

In the early stages of the pandemic, the public policy discussion in developing countries revolved around finding a balance between confinement and adverse economic effects. In the studies of Guedegbe et al (2023) and Manda (2023), implemented in Nigeria and Zambia, respectively, small-scale agriculture benefited in unexpected ways from confinement due to the increased availability of labor to work on the farm and the orientation of markets toward consumption of domestic products. However, in the long term, these confinement policies may imply a reduction in children's education and less access to training for farmers, which could result in lower incomes (OCDE 2020; Guedegbe et al. 2023).

The positive impact of spending more time with the family was mentioned often by farmers (Fig. 2) as urbanization and education rates are high in Ecuador, and many children of cocoa farmers leave the countryside in search of better job opportunities in the cities. This trend is evidenced by the significant decrease in Ecuadorian rural population from 84% in 1950 to 37% in 2010, with rural–urban migration as a determining factor (INEC 2015). The decrease in the rural population due to migration abroad, mainly to the USA, is another factor that has influenced the disintegration of several rural families, a dynamic that has been accentuated during the economic crises that Ecuador has experienced, mainly since the 1990s (Herrera 2022).

In the previous economic crisis, the rural–urban migration in Ecuador had been accentuated by the lack of opportunities in the countryside, mainly for the young population. However, because of the pandemic, many people returned to the countryside in search of food and protection from COVID-19, influencing the configuration of the peasant family (FIAN Ecuador et al. 2022). After losing their urban jobs due to the COVID-19 restrictions, many returned and generated positive rural impact by opening new businesses in the villages that stimulate the territorial economy. These experiences in Ecuador mirror experiences in African countries, where reverse migration can lead to agricultural development in the long term. This migration phenomenon should also be analyzed considering seasonal or peak labor that may have encouraged migration to rural areas during the pandemic (Nolte et al. 2022). The return of migrants to the countryside may also affect pressure on agricultural resources and intensification of agricultural practices (Bista et al. 2022).

The identification of the positive and negative impacts perceived by the farmers under study represents a valuable information input for the planning of policies for post-COVID and climate adaptation. Rasul (2021) identifies parallels between the two crises and their effects on agriculture, considering that both deepen the vulnerability of populations to food insecurity and poverty. Alam et al. (2023) highlighted the importance of incorporating public policy mechanisms that focus on improving the sustainability and resilience of agri-food systems by increasing funds for emergency credit, promoting production diversification, and strengthening farmers' associativity and technical capacities to face crises. The pandemic had the effect of deepening inequalities worldwide, so that if current socioeconomic structures are maintained, future crises will cause severe social disruption (Stevano et al. 2021). Likewise, it is important to strengthen public health programs to face future health crises that could increase the vulnerability of the productive sector, considering the limited response capacity of the Ecuadorian health system during the pandemic (Alava and Guevara 2021; Coral-Almeida et al. 2022).

### Response mobilization by cocoa farmers and intermediaries to counteract the effects of the COVID-19 pandemic differed

Overall, farmers in our sample mobilized more effective responses than intermediaries, specifically when comparing within the seven resource groups (Fig. 4), findings contrary to the initially planned hypothesis. Intermediaries reacted to the COVID-19 pandemic with more and more effective human responses, and farmers rather relied on social responses. The responses implemented on the human resources of the intermediaries were based on the implementation of biosecurity measures for their operational personnel and for the farmers. Furthermore, adjustments to their business model, including the on-farm purchase of cocoa and regulation of reception times and spaces, were implemented. In the case of the farmers, the responses corresponded mainly to the isolation measures established at the national level by government agencies and community actions for the marketing of products.

Sampled farmers mainly activated social responses based on farmer-to-farmer support networks (Fig. 3). This behavior was one of the first actions in several farming communities in Latin America. The response consisted in taking advantage of solidarity among producers, both for the consumption of local products and in the transfer of their products to markets, relying on the resources of the associations present in the territories (Tittonell et al. 2021). Cocoa farmers within our sample showed significant collaborative behavior to cope with the effects of the pandemic, despite the fact that associativity for joint commercialization in the Ecuadorian cocoa sector reaches less than 20% (MAG 2017). Agricultural cooperatives represent a key channel to strengthen strategies that promote the resilience of small farmers, being an important means for the transfer of agricultural technologies and connect with other external actors of development cooperation. Strengthening these associative systems is strategic to contribute to the sustainable development of rural communities (Ingutia 2021).

Public policy should enhance the role of associations through their institutionalization and capacity building of members, since networks based on solidarity among members are key to face crises such as that of COVID-19 (Angaw 2021). In the present case, more than half of the farmers did not feel the support of the government to face the pandemic (Fig. 4), which represents the high importance of establishing public policies to support the producers and strengthen their organizations as a mechanism of collective action that can be activated during crises to contribute to state aid to reach rural communities more efficiently. In this aspect, agricultural associations have a strategic role to play in the creation of food banks to feed vulnerable populations (FAO 2020). During the pandemic, worldwide efforts were made to strengthen food assistance programs as a key public policy to address food insecurity, so it is essential to strengthen the production link of agricultural value chains to ensure the viability of these policies (FAO 2021; Headrick et al. 2022).

Important responses for farmers in the identity group were the consumption of traditional medicine and greater production of subsistence crops. The consumption of traditional medicine and self-protection emerged as alternatives in several rural areas of Ecuador in response to the shortage of medicines in public health centers and the poor condition of roads that affected the movement of people sick with COVID-19 (FIAN Ecuador et al. 2022). Globally, the pandemic revealed the high vulnerability to food insecurity in developing countries. In response to the pandemic, farmers have adopted preferences for local food consumption (Roubík et al. 2022). In subsistence farming systems in China, the COVID-19 confinement increased the consumption of legumes, vegetables, or aquaculture products to improve the body's defenses against the virus (Tian et al. 2022).

### Limitations and future research

Despite providing important insights into the effects of the COVID-19 pandemic on the Ecuadorian cocoa sector, this study has some limitations. A large part of cocoa is still traded outside of sustainable supply chain mechanisms, and our farmer sample was part of a corporate sustainability program. Generalizing to all Ecuadorian cocoa farmers should therefore be done with caution. Furthermore, this analysis did not include a categorization of intermediaries according to the volume of cocoa traded, which would have made it possible to determine the impact and responses to the pandemic considering the size of the business unit. However, this limitation does not affect the validity of the results of the study, since the purpose was to analyze the differences between the two groups of actors, but not within each group, which could be further explored in subsequent studies. The collection of this type of data represented a challenge for the research team due to the secrecy of the business models used by different actors in the value chain, mainly those in the informal economy. Similarly, the characterization of farmers according to their socioeconomic level would have allowed for a deeper analysis of resilience to the pandemic considering access to ex-ante and ex-post risk mitigation strategies.

The different impacts and responses on the resources available to farmers and intermediaries suggest further research on the repercussions of COVID-19 in other value chain links, which will allow for a broader analysis of the vulnerability and resilience of actors to different shocks. On the other hand, the research findings and the analyzed bibliography reveal the importance of further study of the relationship between the impacts and responses of the COVID-19 pandemic and the climate crisis on the actors of agricultural value chains in Ecuador. The negative effects of the pandemic on the resources of the subjects under study encourage a reconfiguration of the public policies of the governments of developing countries that should tend to be more inclusive. To contribute to this process, research can focus on identifying successful policies for the management of the pandemic in other countries and their potential application in the local context.

## Conclusions

This study aimed at identifying differences in impacts and responses toward the COVID-19 pandemic between cocoa farmers and intermediaries in Ecuador. Comparing data from 158 cocoa farmers and 52 intermediaries in coastal Ecuador collected in 2021 revealed substantial differences in impacts and responses between the two groups. Our results show that farmers and intermediaries were similarly affected by the pandemic but farmers mobilized more effective responses. However, great differences exist between the two actors when grouping impacts and responses according to respondents' resources. The findings also reveal that the preconditions of access to credit and profit margins in business models have influenced the impacts and responses of stakeholders mainly on financial resources.

In both groups, the greatest impacts were on financial resources, with the decline in cocoa sales being a triggering factor. However, 20% of the intermediaries reported that they could increase their profits during the pandemic, while farmers consistently had to deal with losses. This dynamic reveals the urgent need to strengthen risk mitigation mechanisms based on financial resources, such as access to contingent credit lines, agricultural insurance, and income diversification, especially among farmers, who are considered the most vulnerable link to market shocks. Based on the literature analyzed in this paper regarding the public policies implemented during the pandemic by governments of developed and developing countries, the need to strengthen the criterion of equity became evident, allowing the social inclusion of vulnerable actors in the value chain and within each group of actors, considering the different intensity of the impacts identified in the actors under study, primarily in terms of financial resources. Further studies on the resilience of agriculture to the pandemic and the analysis of the distribution of the social benefits of public policies will also be fundamental to face the effects of the climate change.

Faced with the effects of the pandemic, farmers activated social resources based on solidarity to collaboratively address challenges, such as production logistics and access to food, thus revealing the importance of strengthening associativity among farmers as an institutionalized and collective action mechanism to cope with other imminent shocks, such as climate change. Intermediaries responded to the pandemic by mobilizing their human resources by implementing biosecurity measures for the personnel working in their facilities. Additionally, several intermediaries responded by purchasing cocoa directly at the farms, incorporating the corresponding logistics into their business models.

Through its agricultural policies, the State has a preponderant role in providing services that enhance the ex-ante and ex-post strategies that small farmers can access in the face of the different risks that affect agriculture. Actions can focus on (1) greater agility of contingency credits from public banks in the face of shocks, (2) multi-actor coordination of collaborative actions to face natural disasters and/or social crises, (3) potentiation of associative marketing and short circuits, and (4) coordination in the creation of contingency funds in the face of falling commodity prices. The policies promoted by the State must incorporate measures that tend to strengthen the capacities of stakeholders for social organization and sustainable crop management, allowing to protect Ecuador's food security and economy in the face of future health and climate crises. Through their sustainability programs, private sector actors can intervene by promoting the institutional strengthening of farmers' associations in an integrated manner. This includes extension services and generating spaces for community self-organization to face economic, social, and environmental risks.

Although intermediaries have greater economic resources to face crises, which makes them less vulnerable than farmers, it should be considered that they play an important role in mobilizing cocoa to other links in the value chain. Consequently, their resilience can be promoted through a contingency credit program, given that their business model requires significant amounts of financial capital to buy and sell cocoa. In addition, considering the proximity of intermediaries to farmers, the state and private companies can take advantage of this channel to disseminate good agricultural practices programs.

## Data Availability

The data collected in this research and the code used are publically available at: https://figshare.com/s/04c583d9b718af9ca0b0.
